# Supplementary material for: Pyruvate Dehydrogenase Kinase 1 inhibition mediated oxidative phosphorylation enhancement in cartilage promotes osteoarthritis progression
Source: BMC Musculoskelet Disord. 2023 Jul 20;24:597. doi: 10.1186/s12891-023-06585-6 (PMC10357736; doi:10.1186/s12891-023-06585-6)
Supplement: Supplementary file 1 — Additional file 1. [file 12891_2023_6585_MOESM1_ESM.zip › MF.Gsea.1653627316242/GOMF_DEMETHYLASE_ACTIVITY.html]

Details for gene set GOMF\_DEMETHYLASE\_ACTIVITY[GSEA]

|  || Dataset | 55457\_collapsed\_to\_symbols.cls.cls #OA\_versus\_normal.cls.cls #OA\_versus\_normal\_repos |
| Phenotype | cls.cls#OA\_versus\_normal\_repos |
| Upregulated in class | normal |
| GeneSet | GOMF\_DEMETHYLASE\_ACTIVITY |
| Enrichment Score (ES) | -0.44798502 |
| Normalized Enrichment Score (NES) | -1.5656607 |
| Nominal p-value | 0.013986014 |
| FDR q-value | 0.15384299 |
| FWER p-Value | 0.999 |
Table: GSEA Results Summary

  

Fig 1: Enrichment plot: GOMF\_DEMETHYLASE\_ACTIVITY      
 Profile of the Running ES Score & Positions of GeneSet Members on the Rank Ordered List

  

| SYMBOL | TITLE | RANK IN GENE LIST | RANK METRIC SCORE | RUNNING ES | CORE ENRICHMENT || 1 | MMACHC | metabolism of cobalamin associated C [Source:HGNC Symbol;Acc:HGNC:24525] | 642 | 0.608 | 0.0016 | No |
| 2 | RIOX1 | ribosomal oxygenase 1 [Source:HGNC Symbol;Acc:HGNC:20968] | 910 | 0.539 | 0.0265 | No |
| 3 | KDM4D | lysine demethylase 4D [Source:HGNC Symbol;Acc:HGNC:25498] | 1180 | 0.480 | 0.0463 | No |
| 4 | JMJD4 | jumonji domain containing 4 [Source:HGNC Symbol;Acc:HGNC:25724] | 1189 | 0.479 | 0.0862 | No |
| 5 | FTO | FTO alpha-ketoglutarate dependent dioxygenase [Source:HGNC Symbol;Acc:HGNC:24678] | 1314 | 0.456 | 0.1151 | No |
| 6 | KDM8 | lysine demethylase 8 [Source:HGNC Symbol;Acc:HGNC:25840] | 2594 | 0.291 | 0.0404 | No |
| 7 | HR | HR lysine demethylase and nuclear receptor corepressor [Source:HGNC Symbol;Acc:HGNC:5172] | 3356 | 0.216 | -0.0005 | No |
| 8 | ALKBH4 | "alkB homolog 4, lysine demethylase [Source:HGNC Symbol;Acc:HGNC:21900]" | 4264 | 0.131 | -0.0598 | No |
| 9 | ALKBH1 | "alkB homolog 1, histone H2A dioxygenase [Source:HGNC Symbol;Acc:HGNC:17911]" | 4765 | 0.090 | -0.0911 | No |
| 10 | KDM6A | lysine demethylase 6A [Source:HGNC Symbol;Acc:HGNC:12637] | 5069 | 0.067 | -0.1090 | No |
| 11 | PHF2 | PHD finger protein 2 [Source:HGNC Symbol;Acc:HGNC:8920] | 5332 | 0.045 | -0.1255 | No |
| 12 | KDM5B | lysine demethylase 5B [Source:HGNC Symbol;Acc:HGNC:18039] | 5484 | 0.033 | -0.1344 | No |
| 13 | KDM5C | lysine demethylase 5C [Source:HGNC Symbol;Acc:HGNC:11114] | 5737 | 0.012 | -0.1530 | No |
| 14 | KDM3B | lysine demethylase 3B [Source:HGNC Symbol;Acc:HGNC:1337] | 6005 | -0.005 | -0.1733 | No |
| 15 | KDM4B | lysine demethylase 4B [Source:HGNC Symbol;Acc:HGNC:29136] | 6441 | -0.038 | -0.2039 | No |
| 16 | JMJD1C | jumonji domain containing 1C [Source:HGNC Symbol;Acc:HGNC:12313] | 6989 | -0.079 | -0.2397 | No |
| 17 | CYP1A1 | cytochrome P450 family 1 subfamily A member 1 [Source:HGNC Symbol;Acc:HGNC:2595] | 8195 | -0.171 | -0.3189 | No |
| 18 | KDM3A | lysine demethylase 3A [Source:HGNC Symbol;Acc:HGNC:20815] | 8264 | -0.176 | -0.3093 | No |
| 19 | KDM1A | lysine demethylase 1A [Source:HGNC Symbol;Acc:HGNC:29079] | 8608 | -0.202 | -0.3188 | No |
| 20 | CYP51A1 | cytochrome P450 family 51 subfamily A member 1 [Source:HGNC Symbol;Acc:HGNC:2649] | 9598 | -0.283 | -0.3717 | No |
| 21 | KDM4C | lysine demethylase 4C [Source:HGNC Symbol;Acc:HGNC:17071] | 10040 | -0.325 | -0.3784 | No |
| 22 | KDM4A | lysine demethylase 4A [Source:HGNC Symbol;Acc:HGNC:22978] | 10936 | -0.423 | -0.4121 | Yes |
| 23 | KDM5A | lysine demethylase 5A [Source:HGNC Symbol;Acc:HGNC:9886] | 11097 | -0.443 | -0.3871 | Yes |
| 24 | RIOX2 | ribosomal oxygenase 2 [Source:HGNC Symbol;Acc:HGNC:19441] | 11444 | -0.495 | -0.3720 | Yes |
| 25 | PHF8 | PHD finger protein 8 [Source:HGNC Symbol;Acc:HGNC:20672] | 11592 | -0.514 | -0.3399 | Yes |
| 26 | CYP1A2 | cytochrome P450 family 1 subfamily A member 2 [Source:HGNC Symbol;Acc:HGNC:2596] | 11719 | -0.536 | -0.3043 | Yes |
| 27 | KDM6B | lysine demethylase 6B [Source:HGNC Symbol;Acc:HGNC:29012] | 11835 | -0.556 | -0.2662 | Yes |
| 28 | UTY | "ubiquitously transcribed tetratricopeptide repeat containing, Y-linked [Source:HGNC Symbol;Acc:HGNC:12638]" | 11984 | -0.590 | -0.2277 | Yes |
| 29 | KDM2A | lysine demethylase 2A [Source:HGNC Symbol;Acc:HGNC:13606] | 12011 | -0.595 | -0.1793 | Yes |
| 30 | JARID2 | jumonji and AT-rich interaction domain containing 2 [Source:HGNC Symbol;Acc:HGNC:6196] | 12030 | -0.600 | -0.1299 | Yes |
| 31 | KDM7A | lysine demethylase 7A [Source:HGNC Symbol;Acc:HGNC:22224] | 12390 | -0.704 | -0.0982 | Yes |
| 32 | KDM5D | lysine demethylase 5D [Source:HGNC Symbol;Acc:HGNC:11115] | 12595 | -0.793 | -0.0468 | Yes |
| 33 | JMJD6 | "jumonji domain containing 6, arginine demethylase and lysine hydroxylase [Source:HGNC Symbol;Acc:HGNC:19355]" | 12658 | -0.832 | 0.0188 | Yes |
Table: GSEA details [plain text format]

  

Fig 2: GOMF\_DEMETHYLASE\_ACTIVITY      
 Blue-Pink O' Gram in the Space of the Analyzed GeneSet

  

Fig 3: GOMF\_DEMETHYLASE\_ACTIVITY: Random ES distribution      
 Gene set null distribution of ES for **GOMF\_DEMETHYLASE\_ACTIVITY**
